# Studies on the quality of duck meat sausages during refrigeration

**DOI:** 10.1186/s40064-016-3743-7

**Published:** 2016-12-01

**Authors:** Z. Naveen, B. R. Naik, B. V. Subramanyam, P. M. Reddy

**Affiliations:** 1 Department of Livestock Products Technology, College of Veterinary Science, Sri Venkateswara Veterinary University, Proddatur, Andhra Pradesh 516360 India; 2 Department of Veterinary Physiology, College of Veterinary Science, Venkateswara Veterinary University, Proddatur, Andhra Pradesh 516360 India; 3 Instructional Livestock Farming Complex, College of Veterinary Science, Sri Venkateswara Veterinary University, Proddatur, Andhra Pradesh 516360 India

**Keywords:** Duck meat, Sausages, Refrigeration, Cooking loss, Emulsion stability, 2-TBA values, Percent moisture, Crude protein, Ether extract

## Abstract

Duck farming is on the raise in the current scenario, but processed products from duck meat are still uncommon to find. Investigating the duck meat qualities during storage will provide information to enhance duck meat utilization. Development of ready-to-eat and ready-to-cook duck meat products is expected to increase and improve non-chicken meat-based protein. The Study was aimed to evaluate the changes in quality characteristics of duck meat sausages preserved by refrigeration (7 ± 1 °C). Duck meat sausages were prepared by utilizing raw and partially cooked duck meat with addition of soy flour at 10% level as a binder. Different quality characteristics like physical and chemical characteristics, proximate composition, and organoleptic characteristics were evaluated. Cooking loss of partially cooked meat sausages was lower than raw duck meat sausages, whereas emulsion stability and 2-thiobarbituric acid (TBA) values of raw duck meat sausages were lesser than partially cooked meat sausages. Cooking loss and emulsion stability decreased in both types of meat sausages, while, 2-TBA values increased as refrigerated storage progressed for 14 days. Percent moisture content of raw duck meat sausages was higher than partially cooked meat sausages, which decreased in both types of meat sausages as refrigerated storage progressed for 14 days. Percent crude protein (CP) and percent ether extract (EE) content of partially cooked duck meat sausages were higher than raw duck meat sausages. Regardless of type of meat used, refrigerated storage of sausages increased CP and EE up to 10th day but decreased upon further storage up to 14th day. Organoleptic scores for raw duck meat sausages were higher than partially cooked duck meat sausages and all the scores decreased with an increase in the storage period. However the scores were within the acceptable limits. The findings prove that, duck meat can be effectively acclaimed as an alternative avenue to meet the escalating protein demand in the form of ready-to-eat product. The quality of sausages is also retained during refrigerated storage.

## Background

Meat and meat products are naturally enriched nutritionally with protein, fat, minerals and vitamins and is conventionally an essential part of the diet (Cosgrove et al. 2005). Meat has emerged as a mass consumer artifact throughout the world and the industrialized Western countries recorded highest consumption rates. A significant percentage of the recommended dietary allowances for proteins, vitamins-B, magnesium, iron and zinc are contributed by meat (Pearson and Brooks 1978). The eating habits of present day health conscious consumer had a dramatic and progressive shift toward foods including meat products with decreased levels of fat, salt, cholesterol and caloric content as well as enriched with dietary fibre (Yang et al. 2007).

Duck is still very famous and is in vigorous demands in many areas of the world, especially in Asia. Ducks come second to chicken for the production of eggs in India. They are predominantly reared for laying purpose. Spent and culled ducks are marketed after 3–4 laying years. Such duck meat intended for human consumption has less juiciness, more toughness, less palatability which are the concealed reasons for unacceptability of the duck meat by the consumers, though there is no substantial decline in its nutritive value with escalation in age (De 2001). Comminuted, emulsion type, value added meat products can be prepared from this desi duck meat to enhance their acceptability. Moreover, processing of duck meat is more critical to provide variety of duck meat products to consumer so that demand and marketability can be augmented. Comminuted emulsion products permit inclusion of edible by-products such as skin, gizzard and heart from the spent birds, which would suffice to reduce the cost of the product and improve the yield and quality of finished product.

Emulsion based product technology is the one which can transform meat of low organoleptic value into a highly acceptable product without tenderization. Therefore processing of meat from ducks and spent hens, a specialized product like sausages can be made affordable even to a low income society of under developed and developing countries. The deviations in consumers’ perception towards the fast food are giving impetus to this field. It is more economical to exploit spent ducks for preparing value added meat products by adding non-meat ingredients, curing salts and seasonings. Inclusion of non-meat additives as binders or extenders had been familiar in production of emulsion based meat products. The palatability of meat products is increased by the addition of various fat substitutes. Among non-meat additives used as fat substitutes, fillers, binders or extenders are wheat flour in chicken nuggets (Rao et al. 1997), soy-flour in buffalo meat burgers (Modi et al. 2003), liquid egg and soyprotein in goat meat patties (Gujral et al. 2002), amaranthus and buck wheat proteins in emulsion type products (Bejesano and Corke 1998), gram flour in low fat patties (Reddy and Rao 1997).

Soybean, which plays a vital role as a protein resource for Asian people is a highly nutritious food material that contains well balanced amino acids and desirable fatty acids. Soy proteins being an cheap source of food protein have been used extensively in meat products as a binder for improving yields, as a gelling agent to enhance emulsion stability and as a meat replacement to reduce costs (Lecomte et al. 1993; Rentfrow et al. 2004).

Over the decades, Snack food is one of the fastest budding segments of the food industry, which has amplified significantly (Thakur and Saxena 2000). The market of snack food industry including semi-processed/cooked and ready to eat foods recorded nearly Rs 82.9 billion in 2004 to 2005 and is escalating rapidly with a growth rate of 20% (Sing et al. 2011). Sausage is a food that is prepared from comminuted and seasoned meat and is usually symmetrical shaped. This was done to preserve fresh meat that could not be consumed immediately. Sausages are getting popularity in Indian market specifically as a fast food in big cities.

In India processing of meat was often overlooked with the argument that there is potential market for fresh meat. This misconception needs to be changed for development of processed meats on strong footing. In this connection, the National Food Processing Policy vision document is developed to inspire value added food products for domestic and export markets with a strong emphasis on food quality and safety empowering farmers to comprehend direct benefits of new technology and marketing network and to ensure adequate availability of quality food products for consumers at economic prices.

Hence, the present study has been carried out with an objective to

- Study the quality of duck meat sausages during refrigeration (7 ± 1 °C).

## Methods

Twenty-four desi ducks weighing 2.4–2.8 kg were slaughtered, dressed conventionally duly following the ethical considerations of Animal Welfare Board of India (AWBI) and Committee for the Purpose of Control and Supervision of Experiments on Animals (CPCSEA); and the whole carcasses, weighing 1.2–1.4 kg, were chilled to an internal temp of 4 ± 1 °C prior to deboning. The dressed whole carcasses were de-boned raw, in four separate batches. Another Twenty-four desi ducks were also slaughtered, dressed conventionally in four separate batches and the whole carcasses were partially cooked in a pressure cooker at 1.5 kg/cm^2^ pressure for 5 min, prior to de-boning, which facilitated easy de-boning.

The above formulations were made up to 100 parts by adding seasonings and condiments, which included: common salt, onion ginger garlic paste (raw), spice mixture, and citric acid as per standard recipe.

### Preparation of duck meat sausages

Both the raw and partially cooked, de-boned duck meats obtained from four batches each, were initially minced separately in a meat mincer (SIRMAN model TC 12E), with a 4 mm diameter sieve. The minced meats were then thoroughly mixed with soya bean flour at 10% level as a binder (by replacing 10% of meat with soya bean Flour) and other ground seasonings and condiments (Table 1), to prepare a uniform batter, separately for raw and partially cooked duck meat (four batches each).

**Table 1:** Levels of incorporation of binder by replacing duck meat

Sausage ingredients	Type of duck meat	
	Raw meat	Partially cooked meat
Duck meat (%)	65	65
Soya bean flour (%)	10	10
Vegetable fat (vanaspati) (%)	20	20
Total	95	95

**Fig. 1:**
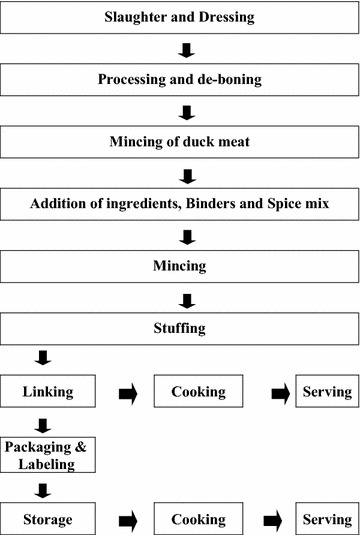
Schematic representation of duck meat sausage preparation

After processing, each formulation was stuffed in sheep casings of 14–18 mm diameter with a sausage stuffer and the sausages were linked to lengths of 1½–2 inches. The schematic representation of preparation of duck meat sausages is presented in Fig. 1. Sausages thus prepared were used for further evaluation, either for laboratory analysis or for sensory evaluation.

Both the raw and partially cooked Duck Meat sausage replicates were divided into four parts weighing 400 g each, wrapped in polythene bags, labeled and kept in refrigerated storage (7 ± 1 °C) in dark. A part of fresh sausages were retained for evaluation on 0 day. The raw and partially cooked Duck Meat sausage samples of the four replicates were withdrawn on 4, 7, 10 and 14th day of dark refrigerated storage and were evaluated for qualitative and organoleptic characters.

The sausages prepared using raw and partially cooked meats in four separate batches separately, were cooked for 15 min, so as to attain an internal temperature of 72 ± 1 °C, fried in a shallow pan, prior to organoleptic evaluation.

The various parameters studied for each sample at different storage periods were

1. Physical characteristics
  - Cooking loss.
  - Emulsion stability.
2. Chemical characteristics
  - 2-Thiobarbituric acid Values
3. Proximate principles
  - Percent moisture.
  - Percent crude protein.
  - Percent ether extract.
4. Organoleptic characteristics

#### Cooking loss

Cooking loss of both raw and partially cooked duck meat sausages were derived (Boles and Swan 1996) by recording the differences in pre and post cooking weights and expressed as percentage.

$${\text{Cooking}}\,\,{\text{loss}}\,\left( \% \right) = \frac{{{\text{Weight}}\,\,{\text{of}}\,\,{\text{sample}}\,\,{\text{before}}\,\,{\text{cooking}} - {\text{weight}}\,\,{\text{of}}\,\,{\text{sample}}\,\,{\text{after}}\,\,{\text{cooking}}}}{{{\text{Weight}}\,\,{\text{of}}\,\,{\text{the}}\,\,{\text{sample}}\,\,{\text{before}}\,\,{\text{cooking}}}} \times 100$$

#### Emulsion stability

Emulsion stability of duck meat sausages was estimated as per Mandal et al. (2001), by taking twenty grams of sausage batter, placed in a polythene bag and heated to 80 °C for 20 min in a water bath. The cookout was drained and the cooked mass weighed to determine the weight loss and expressed as percentage.

$${\text{Emulsion}}\,\,{\text{stability}}\,\left( \% \right) = \frac{{{\text{Weight}}\,\,{\text{of}}\,\,{\text{the}}\,\,{\text{emulsion}}\,\,{\text{after}}\,\,{\text{heating}}}}{{{\text{Weight}}\,\,{\text{of}}\,\,{\text{the}}\,\,{\text{emulsion}}\,\,{\text{before}}\,\,{\text{heating}}}} \times 100$$

#### 2-Thio barbituric acid values

The 2-thiobarbituric acid values of raw and partially cooked sausages at 0 day and refrigerated storage (7 ± 1 °C) samples were determined according to the extraction method described by Wittee et al. (1970), wherein ten grams of sausage batter was blended at full speed for 1.5 min in a mechanical blender, with 25 ml of extracting solution containing 20% tri chloro-acetic acid in 2 M orthophosphoric acid, stored at 4 °C. The resultant solution was transferred quantitatively into a 50 ml volumetric flask and volume is made up to 50 ml with distilled water and mixed thoroughly by shaking. A 25 ml portion of this was filtered through Whatman No. 1 filter paper. 5 ml of the filtrate was transferred to a test tube, followed by addition of 5 ml of 2-thiobarbituric acid (0.005 M in distilled water). The test tube was stoppered and the solution mixed by inversion and kept in dark for 12–15 h, at room temperature. The color developed was measured at 530 nm in a spectrophotometer (EC Digital Spectrophotometer).

The TBA value used to express the result of extraction method was calculated by multiplying the absorbance by the ‘K’ value (5.2) for extraction.

#### Proximate composition

The percent moisture, crude protein and ether extract of raw and partially cooked sausages at 0 day as well as of samples of refrigerated (7 ± 1 °C) storage were estimated according to the standard techniques laid down by AOAC (1990). Moisture content was quantified by oven-drying 10 g duck sausage samples overnight. Crude protein content determination involved Kjeldahl method, where finely minced sausage emulsion was digested using concentrated Sulphuric acid. The Ammonia liberated from the reaction mixture was absorbed in 2% boric acid solution in the distillation section of Kjeldhal apparatus. This solution was titrated against 0.01 N HCl using indicator. Crude Fat content was determined by Soxhlet method using petroleum ether as a solvent.

#### Organoleptic evaluation

Duck meat sausages prepared from raw and partially cooked meat batter were presented for subjective evaluation on 0 day. The samples kept for refrigeration (7 ± 1 °C) were presented for subjective evaluation on 4, 7, 10, and 14th day. The samples were cooked, to attain an internal temperature of 72 ± 1 °C, fried in a shallow pan and served hot to a five member semi-trained panel of habitual meat consumers, for sensory evaluation on a nine point hedonic scale from 1 to 9 (1 = Extremely Poor to 9 = Excellent). All the sensory panelists including male and females, were aged between 35–40 years and belonged to same profession. Equal bite size from each treatment was served in odorless plastic saucers.

The data of results obtained from four replicates of raw and partially cooked meat separately, on 0 day as well as on 4, 7, 10 and 14th day were subjected to appropriate statistical analysis as per the conventional methods of Snedecor and Cochran (1994). The data is represented as Mean ± SE, and is subjected to Two way ANOVA followed by Tukey’s HSD post hoc test—(multiple comparison test).

## Results and discussion

### Cooking loss

The role of Cooking loss in meat and meat products is economically pivotal in commercial food industry, as higher is the cooking loss, fewer will be the servings. The mean cooking losses of duck meat sausages as influenced by refrigerated storage (7 ± 1 °C) for both raw and partially cooked duck meat sausages were presented in Table 2.

**Table 2:** Changes in physical and chemical characteristics of duck meat sausages during refrigeration (7 ± 1 °C)

Parameter	Type of meat	Storage period (days)					Overall mean
		0	4	7	10	14	
Cooking loss (%)	Raw	25.89 ± 1.3	25.24 ± 1.4	24.58 ± 1.4	23.96 ± 1.4	23.05 ± 1.4	24.54^A^ ± 0.6
	Partially cooked	22.15 ± 0.8	22.02 ± 0.9	21.57 ± 0.8	21.02 ± 0.9	20.48 ± 0.8	21.54^B^ ± 0.4
	Overall means	24.20^a^ ± 1.1	24.63^a^ ± 1.2	23.08^a^ ± 1.1	22.49^a^ ± 1.2	21.77^a^ ± 1.1	
Emulsion stability (%)	Raw	93.63 ± 1.0	91.40 ± 0.9	89.64 ± 0.9	86.75 ± 0.8	84.30 ± 0.9	89.14^A^ ± 1.04
	Partially cooked	95.16 ± 0.9	93.64 ± 0.8	92.79 ± 0.9	90.94 ± 0.9	89.26 ± 0.8	92.36^B^ ± 1.12
	Overall means	94.39^a^ ± 1.19	92.52^ab^ ± 1.0	91.22^ab^ ± 1.0	88.85^bc^ ± 1.09	86.78^c^ ± 0.95	
2-TBA values (mg mal kg^−1^)	Raw	0.325 ± 0.01	0.390 ± 0.01	0.468 ± 0.04	0.806 ± 0.03	1.105 ± 0.02	0.619^A^ ± 0.07
	Partially cooked	0.429 ± 0.01	0.488 ± 0.01	0.585 ± 0.02	0.767 ± 0.03	0.871 ± 0.03	0.628^A^ ± 0.04
	Overall means	0.377^a^ ± 0.01	0.439^ab^ ± 0.01	0.527^b^ ± 0.03	0.787^c^ ± 0.03	0.988^d^ ± 0.03	

Means bearing the same superscript for each parameter, row-wise, does not differ significantly (P < 0.01)

The overall mean percent cooking loss of duck meat sausages in the present study were comparatively lesser than duck meat (Shawkat Ali et al. 2007). This might be due to the moisture binding effect of Soya Flour added at 10% level as extender. Similar reduction of cooking loss in restructured chicken steaks, with an increase in the level of extender was reported by Bhoyar et al. (1996). While, Reddy and Rao (1996) stated that lower cooking losses upon addition of extenders might be due to optimum absorption of moisture from the emulsion during cooking and also due to the water binding capacity of the respective flours. The lower cooking losses of duck meat sausages with addition of extenders recorded in the present study corroborated well with the findings of Álvarez and Barbut (2013), who established that, increasing the level oats, in cooked meat batters resulted in a significant decrease in cooking losses. Santhi and Kalaikannan (2014) also inferred that the cooking yield was significantly higher, thereby lesser cooking losses in the nuggets containing oat flour.

The mean percent cooking loss of duck meat sausages containing partially cooked duck meat was significantly (P < 0.01) lower than that of raw duck meat sausages, irrespective of storage period (Table 2). The lesser mean percent cooking losses of partially cooked duck meat sausages might be due to initial loss of moisture in partially cooked meat. Similar to the present finding, Vijayalakhmi (1995) in chicken sausages, Rao (1997) in chicken meat loaves and Devalakshmi (2002) in chicken meat chips also obtained a lesser cooking losses in cooked meat added products.

The percent cooking loss of duck meat sausages decreased non-significantly upon refrigerated storage for 14 days regardless of type of meat used, which might be due to loss of moisture in the form of drip loss. Ismail et al. (2014) also opined that refrigerated storage of duck sausages decreased the cooking loss. The present findings are in conformation with Rajani et al. (2007) who reported that the cooking loss of refrigerated chicken emulsions decreased with the progression of storage period, thereby increasing the cooking yield.

### Emulsion stability

The emulsion stability predicts the shelf life of any meat product. The variations in mean emulsion stability of duck meat sausages affected by type of meat and refrigerated storage (7 ± 1 °C) were shown in Table 2. The higher emulsion stability observed in the present study is mainly due to binding of moisture or fat by soya flour added into the product as extender. The higher emulsion stability may ostensibly due to gelation of starch which enhances the emulsion binding of proteins (Puolanne and Puusunen 1983). Similar higher emulsion stability was observed by Nag et al. (1998) in chicken nuggets with increasing levels of extender, Dushyanthan et al. (2008) in buffalo meat nuggets utilizing different binders. Govind et al. (2013) found that Emu meat sausages with oat flour and corn flour recorded significantly (P < 0.05) higher emulsion stability.

The mean emulsion stability of partially cooked duck meat sausages were significantly (P < 0.01) higher than that of raw duck meat sausages, irrespective of storage periods. These findings are congruent with the findings of Rao (1997) in chicken meat loves and Devalakshmi (2002) in chicken meat chips.

A significant (P < 0.01) decrease in mean emulsion stability of duck meat sausages was observed during refrigerated storage up to 14 days. The results were comparable with the findings of Satyaprasad Reddy (1996) in mutton sausages, Rao and Reddy (2000) in chicken meat loaves and Rajani et al. (2007) in chicken emulsion, who reported a decrease in mean emulsion stability of the products during refrigerated storage.

### 2-Thio barbituric acid values

Estimation of oxidative rancidity is one of the methods to assess the stability of any food product containing lipids. 2-thiobarbituric acid (TBA) assay measures the quantity of malonaldehyde, as it is one of the indicator for measuring oxidative rancidity, which is an oxidative breakdown product formed mainly from peroxidised polyunsaturated fatty acids (Sinhuber and Yu 1977).

Irrespective of storage period, partially cooked duck meat sausages had non-significantly higher overall mean TBA values than raw duck meat sausages in refrigerated storage, at every storage interval. A higher degree of oxidation in partially cooked meat than raw meat was reported by Rao (1997) in cooked chicken meat loaves and Devalakshmi (2002) in cooked chicken meat chips, who observed a higher TBA values in cooked meat products.

Regardless of type of meat used, the overall mean TBA values of duck meat sausages increased significantly (P < 0.01) as refrigerated storage progressed to 14 days. The increase in TBA values might be due to oxidation of fatty acids during storage. Unsaturated fatty acids of the sausages underwent a process of oxidative changes during storage resulting into such increment of TBA value with advancement of storage period. Bhattacharyya et al. (2013) in Duck sausages, Ilayabharathi et al. (2012) in spent chicken sausages, and Biswas et al. (2011) in Duck Patties, also noticed an increase in TBA values upon storage.

### Moisture

Moisture content of emulsion sausages relates to the quality and shelf life of the product. The duck meat sausage formulation in the present study, with 10% Soya Bean Flour added as a binder has lesser moisture percent. This might be due to low moisture content of the binder added. Reduction in moisture content upon addition of extender has been demonstrated by Cosenza et al. (2003) in cabrito smoked sausages. Serdaroglu (2006) documented a decreased moisture content in raw beef patties as a result of the addition of oat flour but increases the moisture content in cooked patties.

Regardless of the storage period, the percent moisture of raw duck meat sausages was significantly higher (P < 0.01) than partially cooked duck meat sausages (Table 3). This is evidently due to loss of moisture during partial cooking. Similarly, Singh and Verma (2000) and Lingaiah (1997) also recorded a reduced moisture content in chicken meat patties added with partially cooked meat.

**Table 3:** Changes in proximate composition of duck meat sausages during refrigeration (7 ± 1 °C)

Parameter	Type of meat	Storage period (days)					Overall mean
		0	4	7	10	14	
Moisture	Raw	54.12 ± 1.6	52.43 ± 1.6	50.36 ± 1.6	49.25 ± 1.6	48.04 ± 1.7	50.85^A^ ± 0.7
	Partially cooked	49.31 ± 0.8	47.03 ± 0.9	45.28 ± 1.4	44.18 ± 1.3	43.88 ± 1.0	45.94^B^ ± 0.6
	Overall means	51.72^a^ ± 1.2	49.73^ab^ ± 1.3	47.82^abc^ ± 1.5	46.72^bc^ ± 1.5	45.96^c^ ± 1.4	
Crude protein	Raw	25.43 ± 0.7	26.33 ± 0.6	27.72 ± 0.7	28.43 ± 0.5	28.06 ± 0.6	27.19^A^ ± 0.4
	Partially cooked	27.80 ± 0.6	29.17 ± 0.7	30.02 ± 0.7	30.75 ± 0.8	29.89 ± 0.7	29.53^A^ ± 0.3
	Overall means	26.62^a^ ± 0.7	27.75^abc^ ± 0.7	28.87^bc^ ± 0.7	29.59^c^ ± 0.7	28.98^bc^ ± 0.7	
Ether extract	Raw	20.45 ± 0.8	21.27 ± 0.9	21.95 ± 0.9	22.30 ± 0.9	22.04 ± 0.9	21.61^A^ ± 0.4
	Partially cooked	22.91 ± 0.9	24.10 ± 1.1	24.69 ± 1.0	25.10 ± 1.2	24.77 ± 0.7	24.32^B^ ± 0.5
	Overall means	21.68^a^ ± 0.9	22.69^a^ ± 1.0	23.32^ab^ ± 1.0	23.70^b^ ± 1.1	23.41^a^ ± 0.8	

Means bearing the same superscript for each parameter, row-wise, does not differ significantly (P < 0.01)

The overall mean percent moisture content of duck meat sausages, irrespective of type of meat used, has decreased during refrigerated storage for 14 days. This might be due to loss of drip fluid during storage and also due to evaporation of moisture from meat in chiller (Arief et al. 1989). The decreasing trend of percent moisture during refrigerated storage recorded in the present study is in conformity with the results of Biswas et al. (2011) in Duck Patties, Rao (1997) in chicken meat loaves and Abdolghafour and Saghir (2014) in buffalo meat emulsion sausages kept for refrigerated storage.

### Crude protein

Protein content is indicative of nutritional quality of the product. The higher crude protein content of the duck meat sausages recorded in the present experiment might be due to the protein contributed by the Soya Bean Flour added at 10% level as a binder.

Irrespective of the storage period, partially cooked duck meat sausages yielded significantly higher crude protein content than the raw meat sausages. This can be attributed to the presence of lesser moisture content and consequent increase in dry matter content of partially cooked meat sausages, which in turn, reflected in higher percent crude protein. Serdaroglu (2006) in beef patties, Singh and Verma (2000) in chicken meat patties, recordeda higher percent crude protein content in products added with cooked meat. Devalakshmi (2002) also observed higher percent crude protein in partially cooked samples of meat chips than that of raw meat chips.

Refrigerated storage of duck meat sausages has caused a significant (P < 0.01) increase in mean percent crude protein content of sausages, irrespective of type of meat used, up to 10 days. But further storage up to 14 days has caused a decrease in mean percent crude protein. The progressive and significant increase of crude protein content upon storage up to 10 days might be due to significant decrease in moisture content in the sausages during storage and this decreased moisture content reflected in yielding higher percent crude protein content. Dewi and Ismail (2010) in duck sausages and Vanitha et al. (2013) in mince based products from Catla fish noticed an increase in Crude Protein content as refrigerated storage period increased. Rao and Reddy (2000) also noticed an similar increase in percent crude protein content in chicken meat loaves as the refrigerated storage period increased. The decrease in percent crude protein content during the later stage of storage might be due to protein decomposition in the stored product. This correlates with the concurrent increase pH and decreased water holding capacity of duck meat sausages in refrigeration up to 14 days. These findings are congruent with the observations of Vijayalakhmi (1995) in chicken sausages and Rajkumar et al. (2004) in chevon patties.

### Ether extract

Percent ether extract of duck meat sausages in the present study is relatively higher, which might be attributed to the fact that 20% vegetable fat was added to the recipe.

At every storage interval, the mean percent ether extract of raw duck meat sausages was significantly (P < 0.01) lower than partially cooked duck meat sausages, irrespective of storage period. Similar to crude protein content, the higher percent ether extract in partially cooked duck meat sausages might be due to significant reduction in moisture content in the sausages during storage. In accordance with the present findings, Serdaroglu (2006) detected a higherether extract contentin cooked beef patties compared to raw meat patties. Rao (1991) also noticed similar higher ether extract content in partially cooked chicken meat patties.

Regardless of type of meat, refrigerated storage of duck meat sausages for 10 days has significantly (P < 0.01) increased the mean percent ether extract values which might be due to significant loss of moisture in samples while in storage. Dewi and Ismail (2010) in duck sausages and Vanitha et al. (2013) in mince based products from Catla fish also noticed an increase in Ether Extract content as refrigerated storage period increased. Further storage of sausages up to 14 days has decreased the percent ether extract values, which may be due to oxidation of fats. This observation corresponds to the TBA values recorded during refrigerated storage. These findings are compatible with the observations of Vijayalakhmi (1995) in chicken sausages and Rajkumar et al. (2004) in chevon patties.

### Organoleptic evaluation

The acceptability of meat product can be assessed by the organoleptic evaluation of the product by the consumer. The alterations in the mean organoleptic scores of duck meat sausages as affected by refrigerated storage, for both raw and partially cooked duck meat sausages are presented in Table 4.

**Table 4:** Changes in sensory quality of duck meat sausages during refrigeration (7 ± 1 °C)

Parameter	Type of meat	Storage period (days)					Overall mean
		0	4	7	10	14	
Color	Raw	7.73 ± 0.04	7.02 ± 0.03	6.67 ± 0.04	5.82 ± 0.03	5.09 ± 0.03	6.45^A^ ± 0.3
	Partially cooked	7.24 ± 0.03	6.43 ± 0.04	6.22 ± 0.03	5.68 ± 0.04	5.05 ± 0.04	6.23^B^ ± 0.4
	Overall means	7.49^a^ ± 0.04	6.73^ab^ ± 0.04	6.45^b^ ± 0.04	5.75 ^cd^ ± 0.04	5.07^d^ ± 0.04	
Flavor	Raw	8.03 ± 0.04	7.82 ± 0.03	7.42 ± 0.03	6.93 ± 0.03	6.55 ± 0.04	7.35^A^ ± 0.3
	Partially cooked	7.98 ± 0.03	7.43 ± 0.03	6.87 ± 0.04	6.62 ± 0.04	6.31 ± 0.04	7.04^B^ ± 0.3
	Overall means	8.01^a^ ± 0.04	7.63^b^ ± 0.03	7.15^c^ ± 0.04	6.78^d^ ± 0.04	6.43^de^ ± 0.04	
Juiciness	Raw	7.82 ± 0.06	7.31 ± 0.03	7.14 ± 0.03	6.73 ± 0.03	6.42 ± 0.05	7.08^A^ ± 0.2
	Partially cooked	7.44 ± 0.03	7.02 ± 0.05	6.76 ± 0.03	6.31 ± 0.05	6.17 ± 0.05	6.74^B^ ± 0.2
	Overall means	7.63^a^ ± 0.05	7.17^b^ ± 0.04	6.95^bc^ ± 0.03	6.52 ^cd^ ± 0.04	6.30^d^ ± 0.05	
Tenderness	Raw	7.78 ± 0.04	7.52 ± 0.03	7.14 ± 0.03	6.82 ± 0.03	6.67 ± 0.04	7.19^A^ ± 0.2
	Partially cooked	7.62 ± 0.04	7.58 ± 0.03	7.22 ± 0.03	6.80 ± 0.03	6.53 ± 0.04	7.15^B^ ± 0.2
	Overall means	7.70^a^ ± 0.04	7.55^a^ ± 0.03	7.18^b^ ± 0.03	6.81^c^ ± 0.03	6.60^d^ ± 0.04	
Overall acceptability	Raw	7.93 ± 0.02	7.68 ± 0.02	7.32 ± 0.01	6.84 ± 0.02	6.52 ± 0.03	7.26^A^ ± 0.3
	Partially cooked	7.87 ± 0.03	7.53 ± 0.03	7.28 ± 0.01	6.74 ± 0.01	6.47 ± 0.02	7.17^B^ ± 0.2
	Overall means	7.90^a^ ± 0.03	7.61^b^ ± 0.03	7.30^c^ ± 0.01	6.79^d^ ± 0.01	6.50^e^ ± 0.03	

Means bearing the same superscript for each parameter, row-wise, does not differ significantly (P < 0.01)

Color is the most important factor considered by the consumer while selecting meat or meat product. The final color of the cooked meat product depends upon the pigmentary changes that take place during cooking. The mean color scores of duck meat sausages decreased significantly (P < 0.01) as the refrigerated storage increased to 14 days. This reduction in color scores of stored product might be due to oxidative fading. The gradual decrease in color scores of products stored at refrigeration storage might also be due to pigment and lipid oxidation resulting in non-enzymatic browning.

Flavor in meat and meat products is of complex nature. Usually flavor precursors develop from proteins, fats and sugars present in meat as volatile in nature. Flavor compounds are released during cooking depending upon temperature time and method of cooking. Refrigerated storage of duck meat sausages for 14 days caused a significant (P < 0.01) reduction in mean flavor scores. Fat oxidation in the stored duck meat sausages might have contributed to such reduction in the flavor score. Deterioration of flavor during storage might be also due to microbial growth and oxidative rancidity (Suresh et al. 2003).

The amount of fluids released during chewing of meat and meat products is an important factor in their organoleptic evaluation. The juiciness is reported to be directly related to the quantum of intra muscular fat (Marbling) and moisture content. Refrigerated storage of duck meat sausages for 14 days significantly (P < 0.01) decreased mean juiciness scores compared to fresh sausages, which is evident due to the loss of moisture during storage.

Tenderness of meat and meat products depend upon age, sex, fiber diameter, sarcomere length and connective tissue content of meat. The tenderness scores of fresh duck meat sausages were significantly (P < 0.01) higher than the sausages in refrigeration for 14 days. This decrease in tenderness scores might be due to reduction in moisture content during storage. The gradual decrease in textural scores might be due to release of moisture (Wu et al. 2000) and depletion of fat during storage (Biswas et al. 2011). Changes of other chemical contents such as protein, moisture, fat collagen and pH value during storage might resulted in the change of texture of cooked sausages (Dong et al. 2007). Similar results were presented by Bhat et al. (2011, 2013a) in chicken *seekh kababs*, Bhat et al. (2013b) in chicken meat balls, Kilinc (2009) in anchovy patties and Thomas et al. (2006) in buffalo meat nuggets during refrigerated storage, respectively.

Refrigerated storage for 14 days caused a significant (P < 0.01) decrease of overall acceptability scores of duck meat sausages. Decrease in all the sensory scores viz., color, flavor, juiciness, tenderness during storage had reflected on lower scores for overall acceptability during storage. The present findings confirm the observations of Biswas et al. (2011) in Duck Patties, Bhattacharyya et al. (2013) and Ismail et al. (2014) in Duck Sausages.

At every storage interval, the mean organoleptic scores of duck meat sausages prepared with raw duck meat was significantly (P < 0.05) higher than the sausages prepared with partially cooked duck meat in all the sensory parameters. This might be due to the loss of certain favorable organoleptic characters due to repeated heat treatment during partial cooking. Similar superior sensory scores for partially cooked meat products were substantiated by Singh and Verma (2000) and Lingaiah and Reddy (2001) in chicken meat patties.

## Conclusion

The scope of Duck meat as a potential source of protein supply has been explored. Consumption of duck meat can be enhanced by preparing further processed products, which can be defined as “transformation of raw carcasses into value-added, easy to prepare and convenient foods” like sausages. Raw Duck Meat Sausages had lesser cooking losses and higher organoleptic scores. Partially Cooked Duck Meat Sausages had better emulsion stability, higher 2-TBA values, Crude Protein and Ether Extract values. Both raw and partially cooked duck meat sausages were acceptable up to 14 days of refrigerated storage, as indicated by the sensory scores. All the sensory parameters scored between 7 (=Moderately Good) and 5 (=Fair) on a 9-point Hedonic scale, after 14 days of refrigerated storage.

## Abbreviations

TBA values: 2-thiobarbituric acid values
CP: crude protein
EE: ether extract
HCl: hydrochloric acid
AOAC: Association of Official Analytical Chemists International
ANOVA: analysis of variance
